# Targeted degradation of VEGF with bispecific aptamer-based LYTACs ameliorates pathological retinal angiogenesis

**DOI:** 10.7150/thno.98467

**Published:** 2024-08-19

**Authors:** Ping Zhou, Sai Zhang, Lin Li, Renshuai Zhang, Guizhi Guo, Yufei Zhang, Runa Wang, Miaoyuan Liu, Zhiyi Wang, Huijie Zhao, Guiwen Yang, Songbo Xie, Jie Ran

**Affiliations:** Center for Cell Structure and Function, Shandong Provincial Key Laboratory of Animal Resistance Biology, College of Life Sciences, Shandong Normal University, Jinan 250014, China.

**Keywords:** VEGF, LYTAC, neovascular ocular disease, angiogenesis, aptamer

## Abstract

**Rationale:** Neovascular ocular diseases (NODs) represent the leading cause of visual impairment globally. Despite significant advances in anti-angiogenic therapies targeting vascular endothelial growth factor (VEGF), persistent challenges remain prevalent. As a proof-of-concept study, we herein demonstrate the effectiveness of targeted degradation of VEGF with bispecific aptamer-based lysosome-targeting chimeras (referred to as VED-LYTACs).

**Methods:** VED-LYTACs were constructed with three distinct modules: a mannose-6-phosphate receptor (M6PR)-binding motif containing an M6PR aptamer, a VEGF-binding module with an aptamer targeting VEGF, and a linker essential for bridging and stabilizing the two-aptamer structure. The degradation efficiency of VED-LYTACs via the autophagy-lysosome system was examined using an enzyme-linked immunosorbent assay (ELISA) and immunofluorescence staining. Subsequently, the anti-angiogenic effects of VED-LYTACs were evaluated using *in vitro* wound healing assay, tube formation assay, three-dimensional sprouting assay, and *ex vivo* aortic ring sprouting assay. Finally, the potential therapeutic effects of VED-LYTACs on pathological retinal neovascularization and vascular leakage were tested by employing mouse models of NODs.

**Results:** The engineered VED-LYTACs promote the interaction between M6PR and VEGF, consequently facilitating the translocation and degradation of VEGF through the lysosome. Our data show that treatment with VED-LYTACs significantly suppresses VEGF-induced angiogenic activities both *in vitro* and* ex vivo*. In addition, intravitreal injection of VED-LYTACs remarkably ameliorates abnormal vascular proliferation and leakage in mouse models of NODs.

**Conclusion:** Our findings present a novel strategy for targeting VEGF degradation with an aptamer-based LYTAC system, effectively ameliorating pathological retinal angiogenesis. These results suggest that VED-LYTACs have potential as therapeutic agents for managing NODs.

## Introduction

Neovascular ocular diseases (NODs), characterized by bleeding and scarring caused by the growth of abnormal leaky vessels, have become the leading cause of irreversible vision impairment worldwide 1,2. Diabetic retinopathy (DR), retinopathy of prematurity (ROP), and age-related macular degeneration (AMD) are the most prevalent neovascular ocular diseases, with their prevalence expected to increase significantly in the coming decades 3-6. The intricate mechanisms responsible for driving this neovascularization process arise from a complex pathological interplay involving numerous growth factors and signaling pathways. Among these factors, vascular endothelial growth factor (VEGF) has been identified as one of the most critical components promoting neovascularization, thus contributing to NODs 7. VEGF acts on vascular endothelial cells by engaging with corresponding receptors (VEGFR), leading to the degradation of the basement membrane of blood vessels and changes in vascular permeability, which in turn facilitates the migration and proliferation of intravascular cells. Consequently, this interacts with surrounding cells and participates in the formation of new blood vessels 8. Therefore, anti-angiogenetic therapy with anti-VEGF agents has emerged as the first-line treatment for NODs 9.

The current pharmacological landscape in ophthalmology prominently features four key anti-VEGF agents, bevacizumab, ranibizumab, aflibercept, and conbercept. Both bevacizumab and ranibizumab are designed as recombinant monoclonal antibodies that effectively inhibit isoforms of VEGF-A. Conversely, aflibercept and conbercept, devised as recombinant fusion proteins, function as a “VEGF trap” to capture various forms of VEGF 10,11. Despite the proven efficacy of these drugs in managing NODs, their therapeutic efficiency is limited due to their macromolecular nature, strong hydrophilicity, and consequent poor penetration of the blood-eye barrier 12. This issue is underscored by studies indicating that approximately 30% of patients do not exhibit improved vision after treatment with these anti-VEGF agents 13. Additionally, there are several persistent obstacles related to this therapeutic therapy, including the demand for frequent injections, the soaring costs associated with these medications, and the limited penetration due to the agents' macromolecular properties 14. Therefore, there is an unequivocal urgency for the development of more potent and efficient VEGF-targeting drugs. Innovations of this nature could significantly enhance anti-angiogenic clinical therapies and offer improved treatment outcomes for individuals grappling with NODs.

DNA aptamers, as short single-stranded oligonucleotides, possess high affinity and specificity for target proteins due to their unique three-dimensional structures 15. Similar to monoclonal antibodies, aptamers can function as antagonists to impede the interaction of disease-associated proteins. Moreover, they possess flexible structures that allow them to denature and renature under different thermal conditions without losing their functionality 16. Compared to monoclonal antibodies or protein/peptides, DNA aptamers present several attractive benefits, including low immunogenicity and toxicity, rapid tissue penetration due to their small molecular weight, and ease of cost-effective production. Consequently, they have been extensively applied in fields such as biosensor detection, clinical diagnostics, and therapeutics 17-19. Accumulating results have demonstrated that DNA aptamers targeting VEGF show promise as therapeutic agents for various disorders, including cancers and NODs 20,21. However, the efficacy of DNA aptamers in binding VEGF or inhibiting the interaction between VEGF and its receptor VEGFR is currently limited, primarily attributed to their relatively short half-life and the challenges associated with their delivery, thereby limiting their therapeutic effectiveness 22.

Recent research has highlighted lysosome-targeting chimeras (LYTACs) as emerging tools for the direct degradation of membrane-associated or extracellular proteins 23. This technology offers a promising approach for downregulating target extracellular proteins by binding and activating the cation-independent mannose-6-phosphate receptor (M6PR), a component involved in transporting certain glycosylated proteins to the lysosome 24. Consequently, the internalized protein of interest is efficiently degraded via the lysosomal protein degradation machinery 25-29. Inspired by these recent advancements in LYTAC technology and the considerable merits of DNA aptamers, we sought to innovate in the arena of VEGF degradation therapy. Herein, we describe the development of bispecific aptamer-based VEGF-degrading LYTACs (VED-LYTACs). The engineered VED-LYTACs exhibit high affinity for VEGF and possess robust capabilities for degrading VEGF both *in vivo* and *in vitro*. Our innovative VED-LYTACs diverge from traditional inhibition by degrading the target VEGF rather than merely impeding its interaction with VEGFR. Our VED-LYTACs demonstrate greater effectiveness than bevacizumab in ameliorating the vascular pathologies associated with DR. This strategy may hold significant potential for the development of VEGF-related therapeutics and offer a clinical breakthrough for diseases characterized by elevated VEGF.

## Results

### Construction and characterization of VED-LYTACs

**Figure 1:**
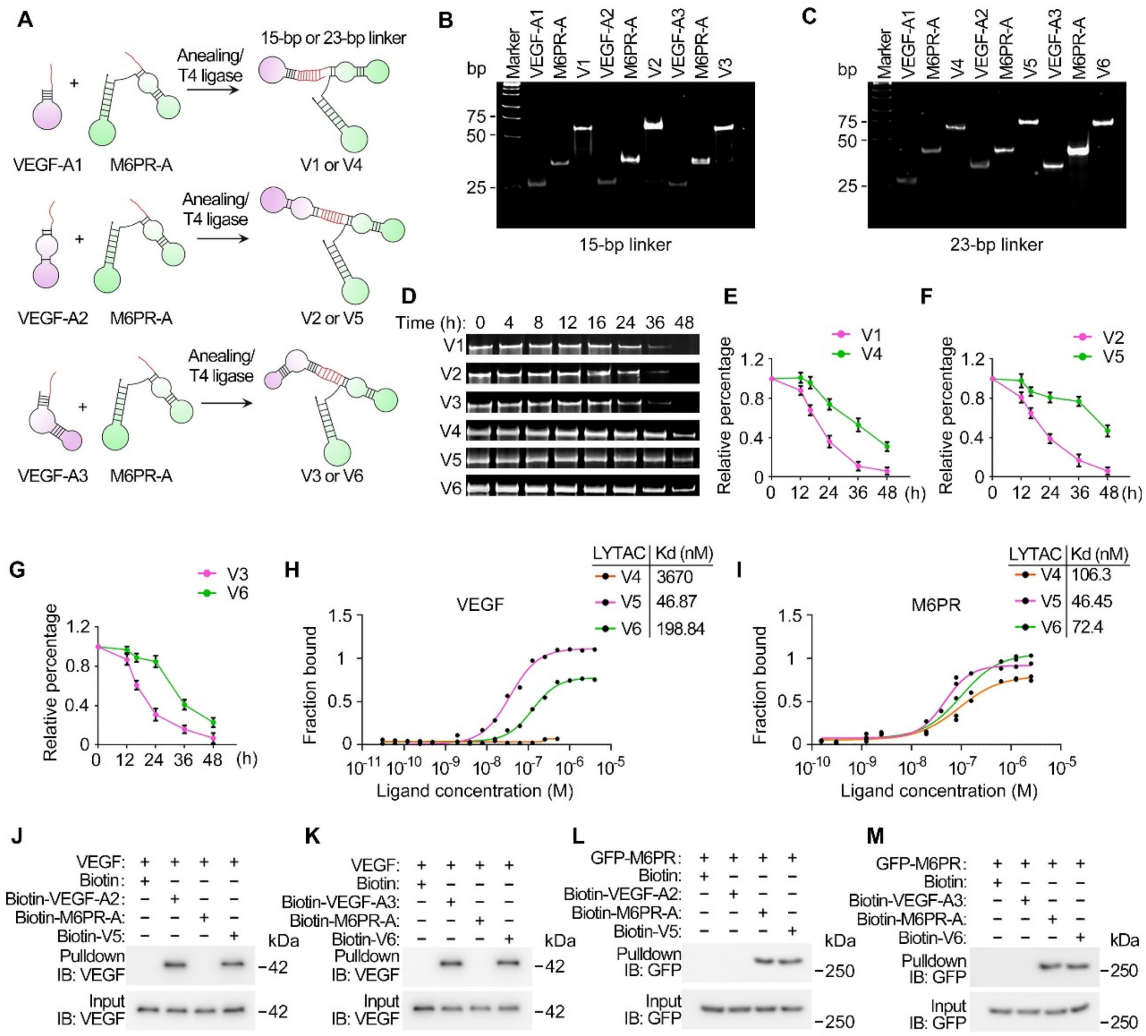
** Characterization of VED-LYTACs. (A)** Schematic representation of the construction of the DNA aptamers-based VED-LYTACs, which contains an M6PR aptamer, an aptamer targeting VEGF, and an additional linker (15-bp or 23-bp double-stranded DNA). The formation of different types of VED-LYTACs (V1 to V6) by changing the design of linkers and aptamers targeting VEGF. **(B and C)** Native polyacrylamide gel analysis of the VED-LYTACs. **(D-G)** Native polyacrylamide gel analysis (D) and quantifications (E-G) of the stability of VED-LYTACs in 10% FBS for different times. **(H and I)** Binding affinity microscale thermophoresis (MST) assay for analyzing the binding affinity of VED-LYTACs with VEGF (H) or M6PR (I) protein. **(J-M)** Streptavidin-pulldown for analysis the interaction between VED-LYTACs (V5 or V6) and VEGF (J and K) or M6PR protein (L and M).

To construct a bispecific aptamer based on LYTAC technology, chimeras were designed to contain three distinct modules: an M6PR-binding motif (M6PR aptamer; M6PR-A) responsible for directing bound molecules to lysosomes for degradation, a VEGF-binding module (VEGF aptamer; VEGF-A) that attaches to VEGF, and a linker essential for bridging and stabilizing the two-aptamer structure (Figure 1A). A 40-nucleotide DNA aptamer with high affinity to M6PR was selected 30, while three DNA aptamers of distinct sequences, designated as VEGF-A1, VEGF-A2, and VEGF-A3, were chosen to target VEGF 20,31-34. Given the significant influence of linker types and lengths on the activities of bispecific aptamers 25,35, 15-base pair (bp) and 23-bp double-stranded DNA (dsDNA) oligos were evaluated as linkers for the VED-LYTACs (Table S1). Pairs of single-stranded linker oligos with complementary sequences were synthesized to contain either M6PR-A or VEGF-A1/2/3, and annealed to obtain six different VED-LYTACs with the abbreviations from V1 to V6 (Figure 1A). It should be noted that the 23-bp linkers of V4, V5, and V6 were covalently ligated using T4 DNA ligase to form the longer dsDNA linker.

To verify the construction of the designed VED-LYTACs, gel electrophoresis was performed to compare the DNA size of VED-LYTACs with their corresponding aptamer components. We found that all the VED-LYTACs were successfully synthesized, as indicated by the DNA molecular weights (Figure 1B-C and Figure S1A). The stability of these chimeras was further assessed by incubating them in cell culture media supplemented with 10% fetal bovine serum to mimic the physiological conditions. Gel electrophoresis demonstrated that the VED-LYTACs with the 23-bp linker (V4, V5, and V6) exhibited a longer half-life compared to V1, V2, and V3 VED-LYTACs (Figure 1D-G).

Given the importance of LYTAC stability for their degradation efficiency, V4, V5, and V6 were selected for further analysis. To test the binding affinities of aptamer chimeras with M6PR and their targeting protein VEGF, microscale thermophoresis (MST), a biophysical assay to quantify the interaction between molecules, was conducted 36. Based on the binding curves and the dissociation constant (Kd) values that reflect the binding affinity between two molecules (a smaller Kd value indicates stronger binding affinity), we observed minimal binding of V4 with VEGF, while V5 exhibited the strongest binding affinity (Figure 1H). Additionally, although all three chimeras were able to bind with M6PR, V5 and V6 showed stronger binding affinities (Figure 1I). To corroborate these findings, *in vitro* streptavidin-pull-down assays were conducted to investigate the interaction of V5 and V6 with VEGF and M6PR. Both chimeras demonstrated robust interactions with VEGF and M6PR (Figure 1J-M). Consequently, these results indicate that V5 and V6 exhibit remarkable stability and binding affinity, suggesting their high potential for effective VEGF degradation functionality.

### VED-LYTACs are effective in targeting VEGF to lysosomes for degradation

**Figure 2:**
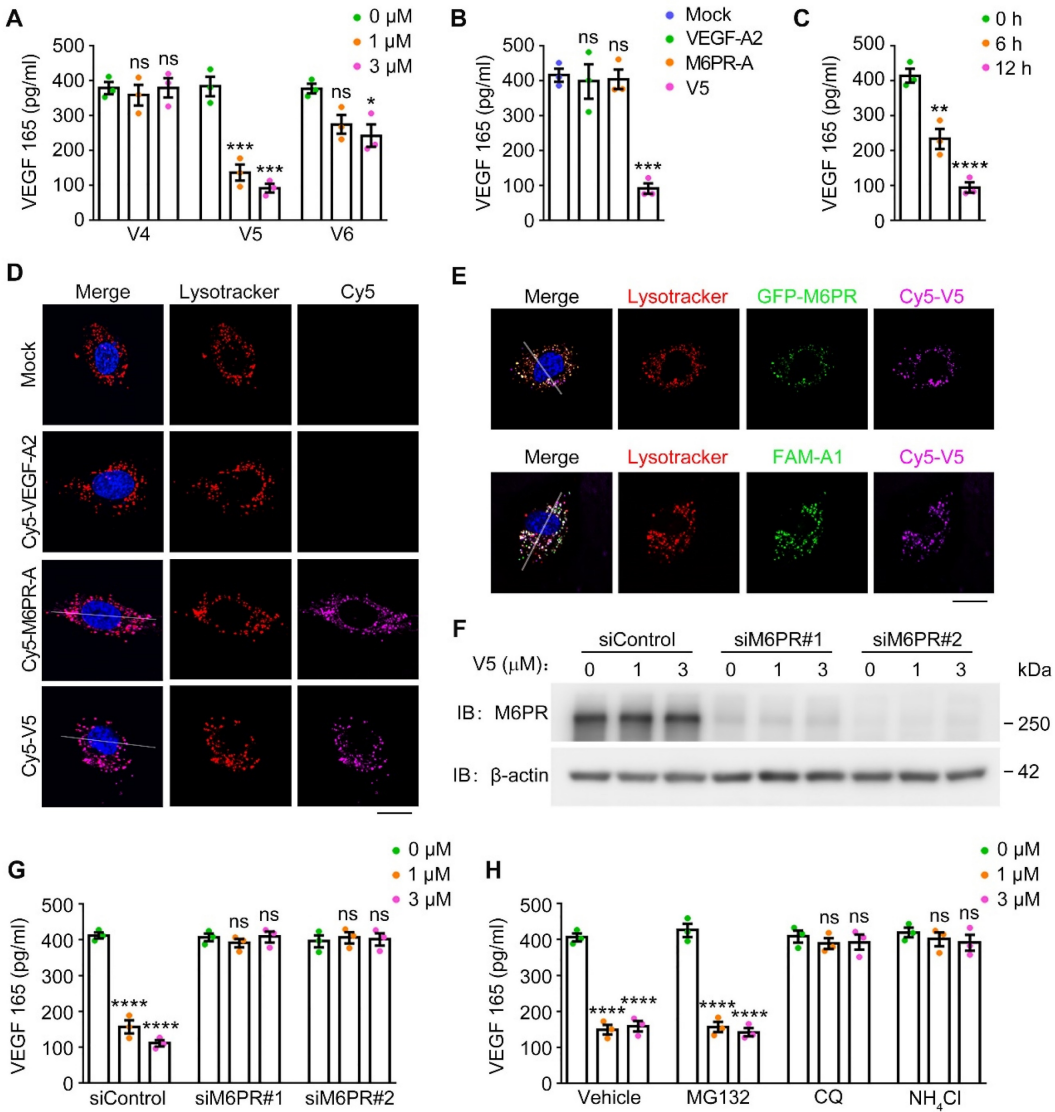
** VED-LYTACs mediate VEGF degration through the autophagy-lysosome system. (A)** ELISA assay for analysis the VEGF level from HUVECs treated with V4, V5 or V6 for 12 h (*n* = 3 independent experiments). **(B)** ELISA assay for analysis the VEGF level from HUVECs treated with the indicated aptamers or V5 for 12 h (*n* = 3 independent experiments). **(C)** ELISA assay for analysis the VEGF level from HUVECs treated with V5 for 6 h or 12 h (*n* = 3 independent experiments).** (D and E)** Immunofluorescence images for lysosome colocalization analysis in HUVECs treated with individual aptamers or V5. Scale bars, 10 µm. **(F)** Immunoblot analysis of M6PR and β-actin in HUVECs transfected with control or M6PR siRNAs and treated with different concentrations of V5. **(G)** ELISA assay for analysis the VEGF level from HUVECs transfected with control or M6PR siRNAs and treated with different concentrations of V5 (*n* = 3 independent experiments). **(H)** ELISA assay for analysis the VEGF level from HUVECs treated with different concentrations of V5, and together with MG132 (5 mM), chloroquine (CQ, 10 mM), or NH_4_Cl (20 mM), or the vehicle (control) for 12 h (*n* = 3 independent experiments). Data are presented as mean ± SEM. *p < 0.05, **p < 0.01, ***p < 0.001 ****p < 0.0001; ns, not significant.

To investigate the potential of VED-LYTACs in reducing VEGF levels, we performed an enzyme-linked immunosorbent assay (ELISA) to examine the effects of the VED-LYTACs treatment on the VEGF secretion of human umbilical vein endothelial cells (HUVECs). Our findings revealed a significant decrease in VEGF levels following treatment with V5, which also occurred in a dose-dependent manner (Figure 2A). Conversely, V6 exhibited a less pronounced ability to degrade VEGF (Figure 2A), which was likely attributed to the weaker binding affinity of V6 with VEGF and M6PR.

To further validate the specificity of V5-mediated VEGF degradation, we treated HUVECs with VEGF-A2, M6PR-A, or V5, and assessed the impacts on VEGF levels. Our results demonstrated that only V5 had the ability to efficiently degrade VEGF but not the individual aptamers (VEGF-A2 and M6PR-A) (Figure 2B). This suggests that the combination of VEGF-A2 and M6PR-A within the V5 molecule is essential for its VEGF degradation activity. Furthermore, we observed a time-dependent degradation effect of V5 on VEGF levels (Figure 2C), further underlining V5 as possessing the most potent VEGF degradation ability among these chimeras. Additionally, a similar VEGF degradation effect was observed in mouse brain microvascular endothelial (bEND.3) cells, indicating that V5 can mediate VEGF degradation in both human and mouse cells (Figure S1B-C).

We then investigated the intracellular localization of V5 and individual aptamers and found that V5 and M6PR-A, but not VEGF-A2, were both effectively internalized into lysosomes, as evident by significant colocalization with a lysosomal tracker (Figure 2D and Figure S1D-E). Additionally, fluorescence microscopy images revealed that VEGF and M6PR colocalize with V5 within lysosomes, suggesting that these three components can form a ternary complex that is trafficked to the lysosome (Figure 2E and Figure S1F-G). Supporting these results, we observed that depletion of M6PR compromised the VEGF degradation capability of V5 (Figure 2F-G), suggesting that the efficient targeting of VEGF to lysosomes by V5 is mediated through the M6P-dependent internalization.

To elucidate the pathway through which V5 mediates VEGF degradation, HUVECs were subjected to the treatment of various inhibitors targeting distinct cellular degradation systems. Specifically, MG132 37, and chloroquine (CQ) and ammonium chloride (NH_4_Cl) 38,39, were respectively utilized to inhibit the proteasome and autophagy-lysosome degradation pathway. Our results revealed that treatment with CQ and NH_4_Cl significantly mitigated the VEGF diminution induced by V5 but not with MG132, suggesting the involvement of the autophagy-lysosome system in the VED-LYTAC mediated degradation (Figure 2H). Collectively, these results demonstrate that the significant reduction in VEGF levels observed with V5 treatment can be attributed to its selective targeting of VEGF to lysosomes for internalization and subsequent degradation.

### VED-LYTACs exhibit anti-angiogenic ability both *in vitro* and* ex vivo*

Given the important role of VEGF in angiogenesis and the observed efficient degradation of VEGF mediated by V5, we thus performed a series of *in vitro* angiogenesis assays to delineate the effects of V5 on the angiogenic capabilities of endothelial cells.

**Figure 3:**
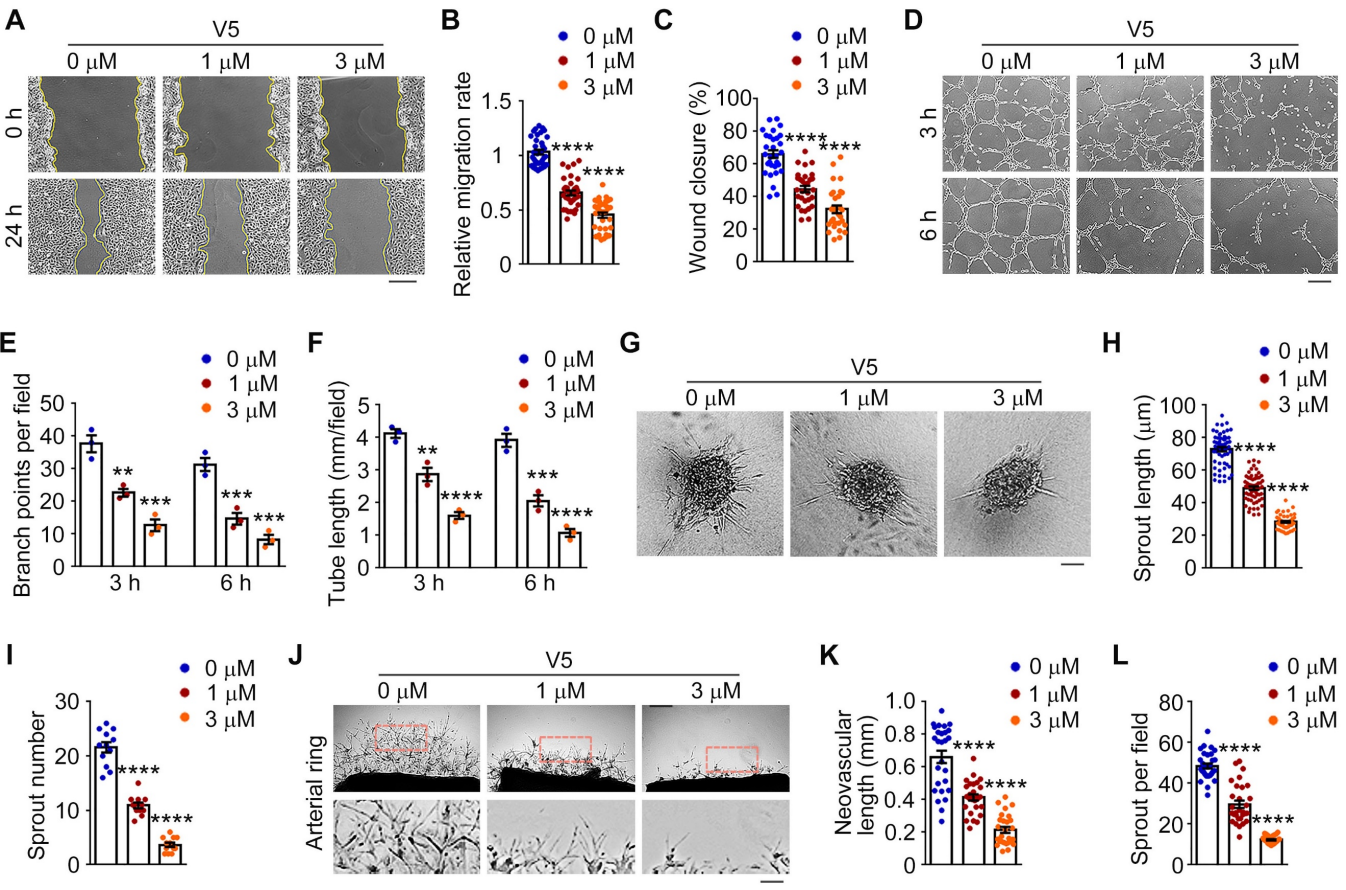
** VED-LYTACs suppress VEGF-dependent angiogenic effects both *in vitro* and *ex vivo*. (A-C)** Representative images (A) and quantifications of the relative migration rate (B, *n* = 30 areas from 3 independent experiments) and percentage of wound closure (C, *n* = 30 areas from 3 independent experiments) for the scratch migration assay in HUVECs treated with different concentrations of V5. Scale bar, 80 µm. **(D-F)** Representative images (D) and quantifications of the branch points (E, *n* = 3 independent experiments) and the tube length (F, *n* = 3 independent experiments) for tube formation assay in HUVECs treated with different concentrations of V5. Scale bar, 200 µm. **(G-I)** Representative images (G) and quantifications of the total sprout length (H, *n* = 50-80 sprouts from 3 independent experiments) and numbers (I, *n* =12 spheroids from 3 independent experiments) for sprouting assay from HUVECs treated with different concentrations of V5. Scale bar, 30 µm. **(J-L)** Representative images (J) and quantifications of the neovascular length (K, *n* =30 fields from 3 independent experiments) and the sprout numbers per field (L, *n* =30 fields from 3 independent experiments) for sprouting assay from mice aortic ring treated with different concentrations of V5. Scale bar, 300 µm. Data are presented as mean ± SEM. **p < 0.01, ***p < 0.001, ****p < 0.0001.

Initially, we investigated the impact of V5 on endothelial cell migration, a critical process of blood vessel formation 40. Under normal conditions, following a scratch, endothelial cells nearly close these gaps within 24 h. Yet, in the presence of V5, these cells showed a substantial decrease in cell migration speed, resulting in a significantly slower wound closure rate, both in HUVEC and bEND.3 cells (Figure 3A-C and Figure S2A-C). Additionally, we evaluated the effects of V5 on the tube formation capacity of endothelial cells, another key aspect of angiogenesis 41. Interestingly, by measuring branch points and total tube length, we found that V5 also inhibits endothelial cells from forming tubular structures (Figure 3D-F). When seeded into the collagen matrix, HUVEC spheroids can extend tube-like sprouts into the collagen matrix. However, sprouting was remarkably inhibited in HUVECs treated with V5 (Figure 3G-I).

To confirm the specific inhibitory role of V5 in angiogenesis, we employed the mouse aortic ring assay as an *ex vivo* model to evaluate sprouting angiogenesis 42. We found that V5 significantly inhibited the formation of vascular networks in aortic rings, confirming its effect on vasculature generation inhibition (Figure 3J-L). Collectively, these results indicated that V5 inhibits angiogenesis both *in vitro* and *ex vivo*.

Additionally, considering that aptamers can function as antagonists to impede the interaction of disease-associated proteins, we subsequently analyzed whether the A2 aptamer, a component of V5, possesses comparable inhibitory effects on angiogenesis. The results indicated that, at equivalent concentrations and treatment durations, the A2 aptamer exhibits significantly less efficacy in inhibiting angiogenesis compared to V5 (Figure S3A-L). This suggests that the effectiveness of V5 likely relies on its multifunctional and structural interactions.

### VED-LYTACs show excellent biosafety and anti-angiogenic ability* in vivo*

Prior to examining the effectiveness of treatment in animals, the *in vivo* biosafety of LYTACs was assessed. To this end, normal animals received intravitreal injections of PBS and V5. One-week post-injection, retinal structure and function were examined. The whole retinal histology was examined by microscopic analysis of hematoxylin and eosin (H&E)-stained sections. The results indicated that the retinal structure layers remained intact without any pathological abnormalities (Figure S4A-B). Subsequently, retinal function was evaluated by full-field electroretinography (ERG), which measures the mass electrical response of the retina to photic stimulation 43. Our findings indicated no significant changes in the the a-wave of the response, reflecting the general physiological activity of photoreceptors in the outer retina, or the b-wave reflecting the inner layer activity of ON bipolar cells and Müller cells in the V5-injected mice, compared to the controls (Figure S4C-E). These results demonstrated the excellent biosafety of V5 in mouse retinas.

**Figure 4:**
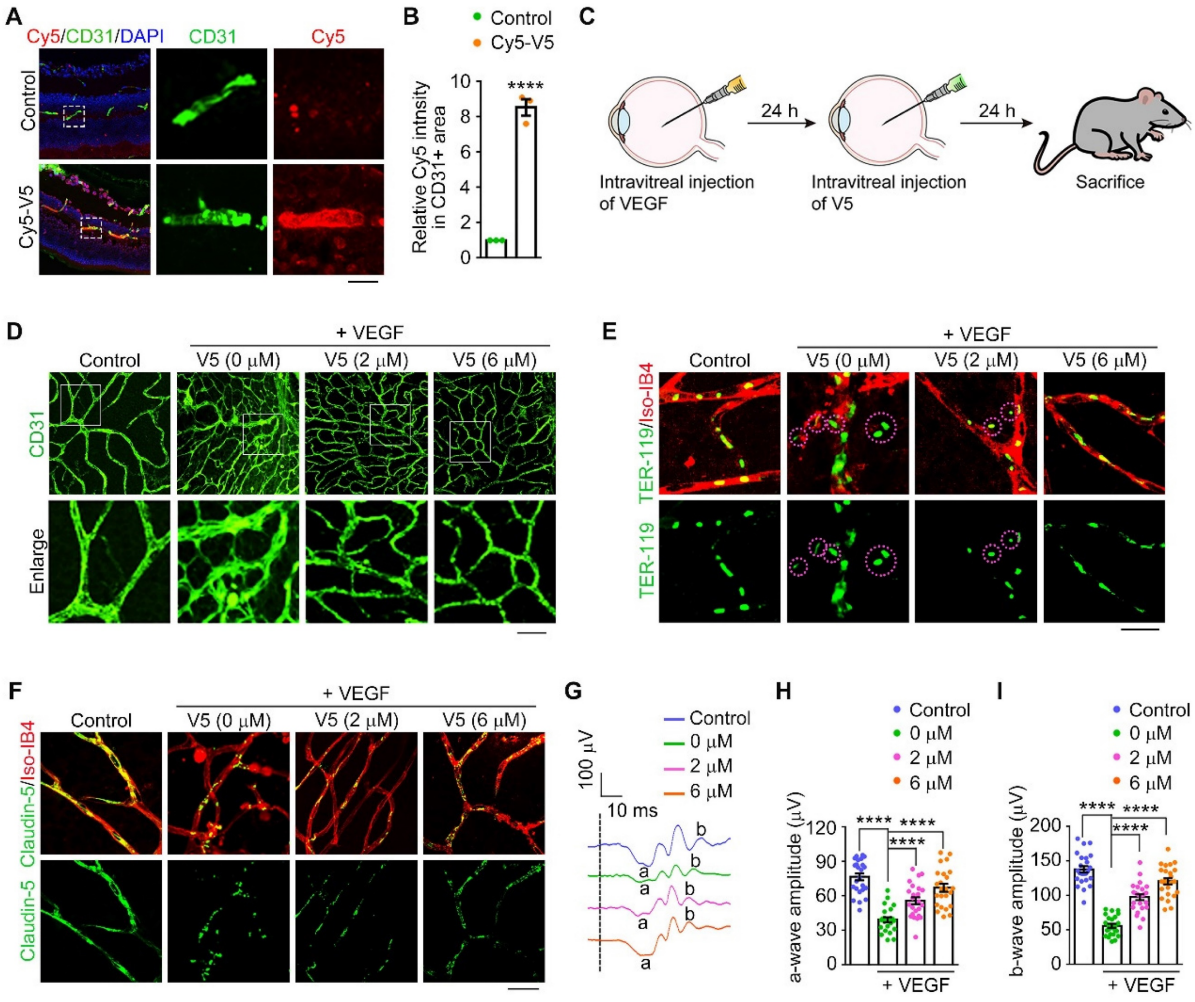
** VED-LYTACs suppress VEGF-induced retinal neovascularization and vascular leakage in mice. (A and B)** Immunofluorescence images (A) and quantification of fluorescence intensity of Cy5-V5 in CD31 positive area (B, *n* = 3 independent experiments) in retinas injected with Cy5 labeled V5 or an unordered DNA sequence as control, and stained with anti-CD31 to label the retinal vessel. Scale bar, 50 µm. **(C)** Schematic depiction of V5 administration in VEGF-induced retinal neovascularization model. **(D)** Immunofluorescence images of the retinal vasculature stained with anti-CD31 in retinas injected with VEGF or IgG (control), and together with different concentrations of V5. Scale bar, 30 µm. **(E)** Immunofluorescence images of retinas from control or VEGF-injected mice treated with different concentrations of V5, and stained with antibody against TER-119 and Alexa fluor 568-conjugated Iso-IB4. Purple circles indicate regions of red blood cell leakage. Scale bar, 20 µm. **(F)** Immunofluorescence images of retinas from control or VEGF-injected mice treated with different concentrations of V5, and stained with antibody against claudin-5 and Alexa fluor 568-conjugated Isolectin-IB4 (Iso-IB4). Scale bar, 20 µm. **(G-I)** ERG recordings (G) and measurement of retinal a-wave (H, *n* = 24 eyes from 3 independent experiments) and b-wave (I, *n* = 24 eyes from 3 independent experiments) amplitudes for control and VEGF-injected mice treated with different concentrations of V5 under scotopic conditions at 3 cd s m^-2^ flash intensity. Data are presented as mean ± SEM. ****p < 0.0001.

We subsequently investigated the therapeutic potential of V5 in attenuating pathological angiogenesis. We set out to ascertain the *in vivo* stability and efficacy within murine ocular tissues treated with intravitreal injections of V5 followed by *in vivo* imaging. Remarkably, the V5 molecules remained detectable within the eye for up to 48 h post-injection and retained their VEGF-degrading capability for up to 96 h post-injection (Figure S5A-B). Additionally, immunofluorescence microscopy validated the entry of V5 into the retinal vascular system, attesting to its stability and bioavailability, essential for sustained therapeutic effect (Figure 4A-B).

Elevation of VEGF is a known trigger for vascular proliferative ocular pathologies. To substantiate the beneficial effects of V5 on pathological angiogenesis, we employed a mouse model that recapitulates retinal angiogenesis driven by VEGF overexpression (Figure 4C). Following VEGF induction, an assessment of the vascular network conducted 24 h later showed significant proliferation, confirming VEGF's role in vascular expansion. In stark contrast, V5 administration resulted in a considerable suppression of neovascularization, thereby highlighting its capacity to mitigate the effects of VEGF-driven pathological angiogenesis (Figure 4D and Figure S5C).

Pathological vascular growth is also implicated in compromising the blood-retinal barrier, potentially leading to vascular leakage, hemorrhage, retinal detachment, and ultimately culminating in visual loss or blindness 9. Employing our VEGF-induced retinal angiogenesis model, we assessed vascular leakage by co-immunostaining with anti-TER-119, an erythroid cell marker 44, and Isolectin-IB4 (Iso-IB4), which labels the vascular endothelial cells. The VEGF-elicited vascular leakage was notably mitigated by V5 administration, evidenced by the dramatic reduction in TER-119-positive and Iso-IB4-negative areas (Figure 4E and Figure S5D). Moreover, tight junction integrity, a critical determinant of vascular barrier functionality and permeability, was scrutinized. The enhancement of VEGF disturbed the tight junction network, as indicated by the discontinuous distribution of claudin-5. In striking contrast, treatment with V5 substantively redressed these VEGF-induced junction disruptions (Figure 4F and Figure S5E). Collectively, our findings provide compelling evidence that V5 not only mitigates VEGF-driven angiogenesis but also restores the integrity of the impaired blood-retinal barrier.

Considering our observation that V5 substantially mitigates pathological activity associated with vascular irregularities, it is plausible to speculate that V5 is promising for reversing retinal dysfunction caused by VEGF overexpression. ERG recordings indicated a significant reduction in both the a-wave and the b-wave, by over 50% in the VEGF-injected mice when compared with the control group (Figure 4G-I). However, following treatment with V5, we observed a marked restoration of retinal function in the VEGF overexpression model, suggesting the significant role of V5 in alleviating VEGF overexpression-induced retinal dysfunction. Collectively, these observations support the potential application of V5 as a therapeutic modality for ocular diseases associated with pathological angiogenesis.

### VED-LYTACs display therapeutic effect in a mouse model of oxygen-induced retinopathy

**Figure 5:**
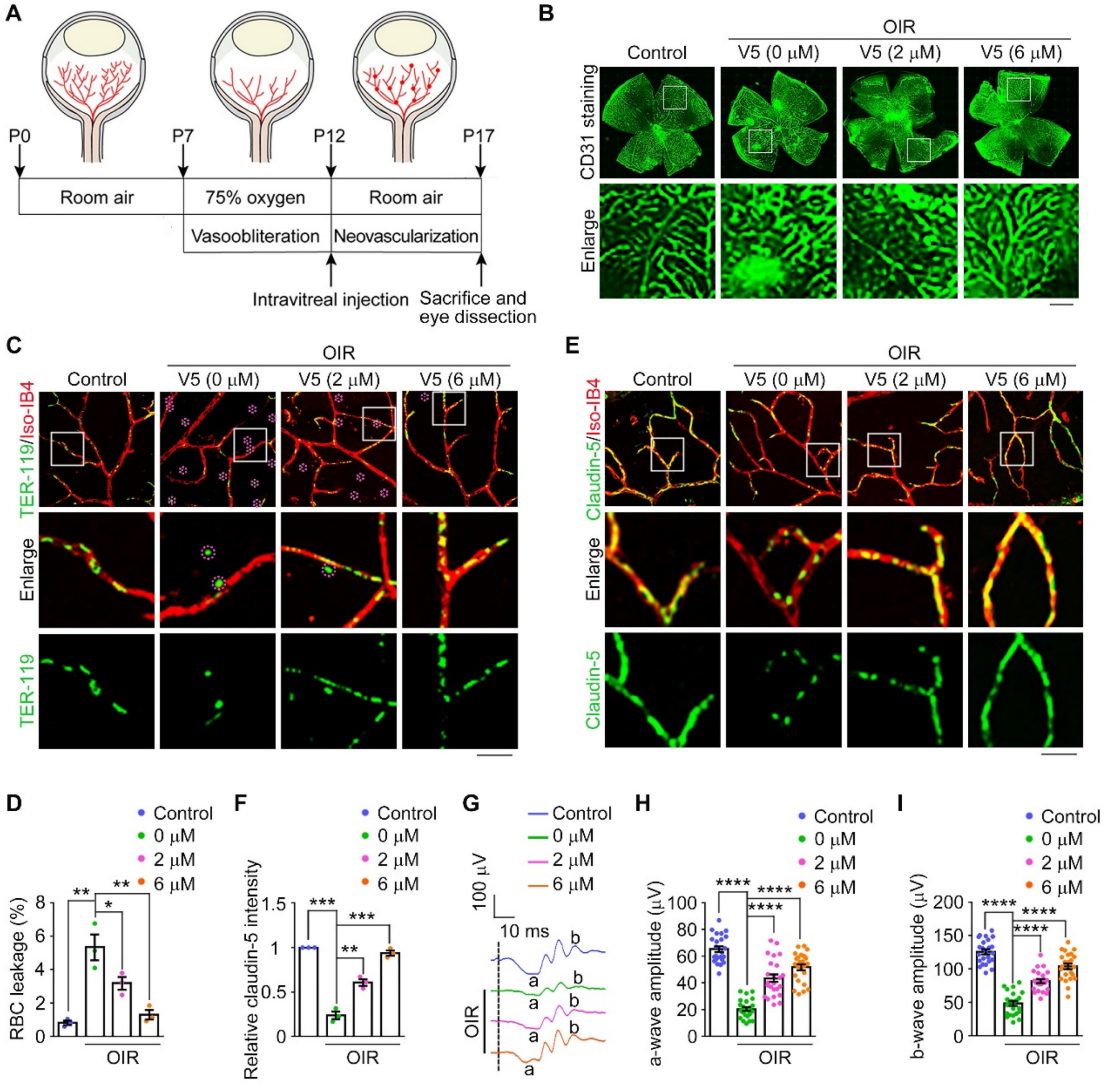
** VED-LYTACs exhibit anti-angiogenic effect in the OIR mouse model. (A)** An illustration of the OIR mouse model. The mouse neonates undergo a hyperoxic (75% oxygen) phase from P7 to P12 and a subsequent relative hypoxic phase (room air). **(B)** Immunofluorescence images of the retinal vasculature stained with anti-CD31 in retinas from control or OIR mice injected with different concentrations of V5. Scale bar, 200 µm. **(C and D)** Immunofluorescence images (C) and quantification of the percentage of RBC leakage (D, *n* = 3 independent experiments) in retinas from control or OIR mice injected with different concentrations of V5, and stained with antibody against TER-119 and Alexa fluor 568-conjugated Iso-IB4. Purple circles indicate regions of red blood cell leakage. Scale bar, 20 µm. **(E and F)** Immunofluorescence images (E) and quantification of the relative claudin-5 intensity (F, *n* = 3 independent experiments) in retinas from control or OIR mice injected with different concentrations of V5, and stained with antibody against claudin-5 and Alexa fluor 568-conjugated Iso-IB4. Scale bar, 20 µm. **(G-I)** ERG recordings (G) and measurement of retinal a-wave (H, *n* = 24 mice from 3 independent experiments) and b-wave (I, *n* = 24 mice from 3 independent experiments) amplitudes for control and OIR mice injected with different concentrations of V5 under scotopic conditions at 3 cd s m^-2^ flash intensity. Data are presented as mean ± SEM. *p < 0.05, **p < 0.01, ***p < 0.001, ****p < 0.0001.

Inspired by the demonstrated efficacy of V5 as an anti-angiogenic agent *in vitro*, *ex vivo*, and *in vivo*, we further explored its therapeutic potential in a mouse model of oxygen-induced retinopathy (OIR) 45. In this model, neonatal mice were subjected to a hyperoxic environment (75% oxygen) from postnatal day 7 to 12 (P7-P12), followed by a phase of relative hypoxia upon cessation of oxygen supplementation (Figure 5A). This mouse model closely mirrors the pathological phenotypes observed in human retinopathy of prematurity, where hyperoxia initially reduces VEGF levels, causing vaso-obliteration, and subsequent hypoxia triggers a compensatory upregulation of VEGF, resulting in pathological neovascularization characterized by the overgrowth of immature vasculature and the formation of extraretinal vascular tufts 46 (Figure 5A and Figure S6A). Consistent with our expectations, we observed elevated VEGF levels in OIR mice. However, treatment with the V5 aptamer significantly reduced VEGF levels in the retinas of these mice, demonstrating V5's capacity for VEGF degradation *in vivo* (Figure S6B). Correspondingly, the pathological vascular proliferation induced by increased VEGF levels in OIR mice was effectively suppressed by V5 (Figure 5B and Figure S6C). This suggests that V5 not only decreases VEGF levels but also ameliorates the resultant aberrant neovascularization, underscoring its potential as a therapeutic agent in conditions characterized by pathological angiogenesis.

As we observed diminished neovascularization in V5-treated OIR mice, we further explored whether V5 could alleviate the VEGF-dependent activities associated with hypoxia-induced angiogenesis, with a particular focus on vascular leakage. With the OIR mice, we monitored hemorrhagic manifestations by detecting leaked erythrocytes (Figure 5C). Remarkably, following V5 intervention, the OIR-induced erythrocyte seepage was significantly mitigated, suggesting that V5 can counteract hypoxia-driven pathogenic processes by stabilizing vascular integrity and curtailing detrimental hemorrhage (Figure 5C-D). Correspondingly, we also discovered that V5 treatment ameliorated the uneven distribution of claudin-5 observed in the OIR-induced mice, a disruption linked to VEGF-mediated vascular permeability (Figure 5E-F). This restoration indicates that V5 not only reduces the overt symptoms of leakage by reinforcing barrier integrity but also tends to mend the underlying endothelial cell junctional disruptions typically exacerbated by elevated VEGF levels.

Our previous studies, along with findings from others, have substantiated that in mice subjected to OIR, the deterioration of vascular architecture precipitated aberrant retinal function 47,48. Indeed, both the a-wave and the b-wave were reduced by over 50% in the OIR mice when compared with the control group (Figure 5G-I). However, following treatment with V5, we observed a substantial restoration of retinal function in the OIR model, suggesting the significant role of V5 in alleviating the OIR-induced retinal dysfunction. Collectively, these results indicate that V5 emerges as a potential pharmacological intervention for OIR and possibly other retinal conditions with similar pathological manifestations.

### VED-LYTACs attenuate pathological retinal neovascularization and vascular leakage in a mouse model of DR

Inspired by the effective alleviation of OIR-induced neovascularization and associated vascular pathologies observed with V5 treatment, we sought to explore its potential therapeutic effects in DR. DR is a prevalent complication of diabetes and a leading cause of blindness in working-aged people 5. The pathology of DR is characterized by early pericyte loss, vascular leakage, formation of acellular capillaries, and late-stage retinal ischemia, which precipitate excessive retinal neovascularization 3. This process is exacerbated by endothelial cell dysfunction triggered by hypoxia and elevated VEGF expression, culminating in vascular leakage and neovascularization.

**Figure 6:**
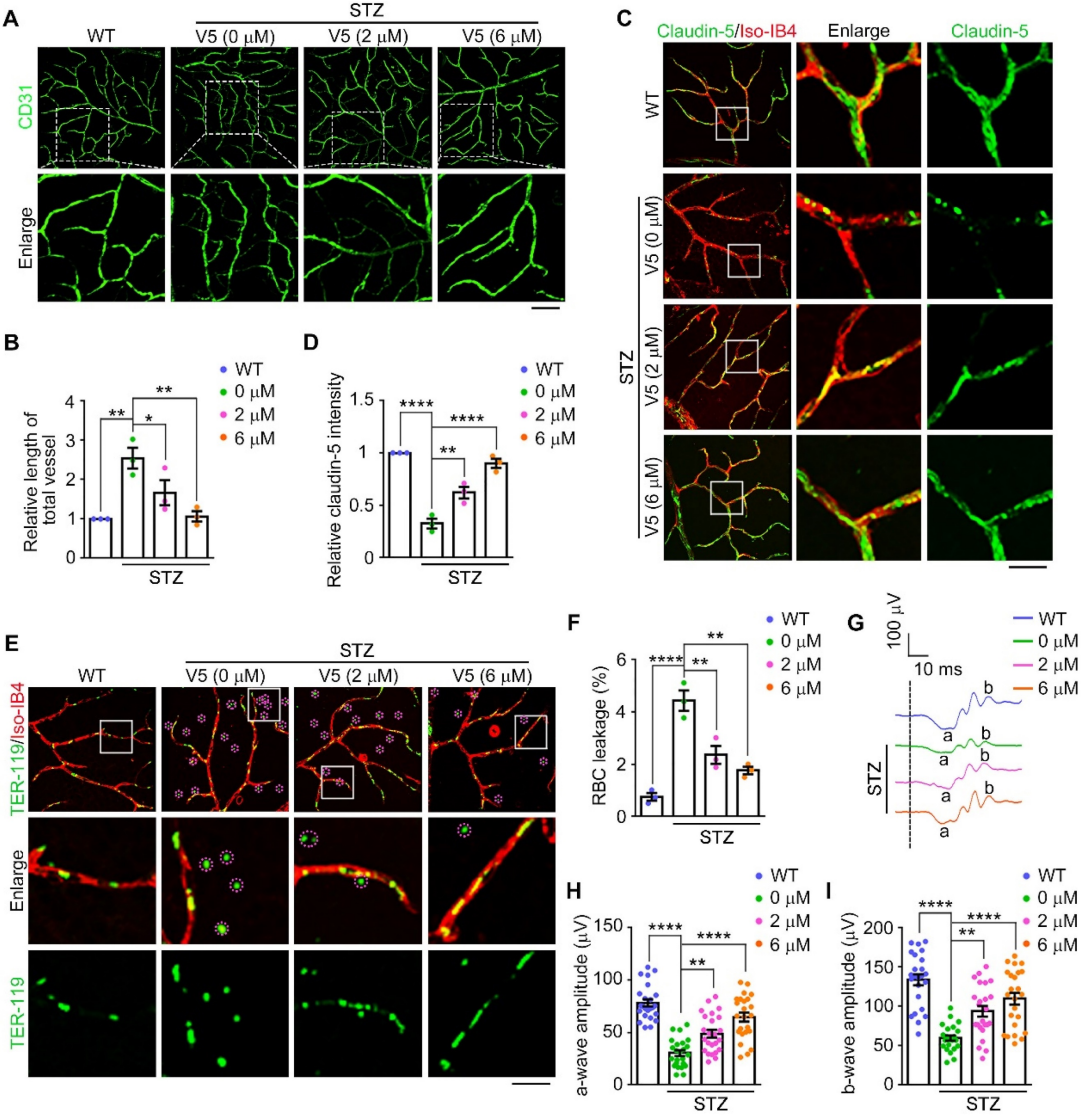
** VED-LYTACs ameliorate pathological retinal neovascularization and vascular leakage in the DR mouse model. (A and B)** Immunofluorescence images (A) and quantification of the relative retinal vessel length (B, *n* = 3 independent experiments) for the retinal vasculature stained with anti-CD31 in retinas from control or STZ-induced diabetic mice treated with different concentrations of V5. Scale bar, 50 µm. **(C and D)** Immunofluorescence images (C) and quantification of the relative claudin-5 intensity (D, *n* = 3 independent experiments) in retinas from control or STZ-induced diabetic mice treated with different concentrations of V5, and stained with antibody against claudin-5 and Alexa fluor 568-conjugated Iso-IB4. Scale bar, 20 µm. **(E and F)** Immunofluorescence images (E) and quantification of the percentage of RBC leakage (F, *n* = 3 independent experiments) in retinas from control or STZ-induced diabetic mice injected with different concentrations of V5, and stained with antibody against TER-119 and Alexa fluor 568-conjugated Iso-IB4. Purple circles indicate regions of red blood cell leakage. Scale bar, 20 µm. **(G-I)** ERG recordings (G) and measurement of retinal a-wave (H, *n* = 24 eyes from 3 independent experiments) and b-wave (I, *n* = 24 eyes from 3 independent experiments) amplitudes for control or STZ-induced diabetic mice injected with different concentrations of V5 under scotopic conditions at 3 cd s m^-2^ flash intensity. Data are presented as mean ± SEM. *p < 0.05, **p < 0.01, ****p < 0.0001.

To determine the role of V5 in pathological retinal neovascularization and vascular leakage, we exploited a streptozotocin (STZ)-induced mouse model of DR, following a well-established procedure 49. Subsequently, the efficacy of V5 as a therapeutic agent was evaluated in this model. Our observations revealed that VEGF levels and formation of diabetes mellitus-induced acellular capillaries were significantly reduced by V5 treatment (Figure 6A-B and Figure S6D). Additionally, the interspersed endothelial cell junction anomalies characteristic of diabetic conditions was mitigated following V5 administration (Figure 6C-D). Concurrently, V5 treatment also resulted in a notable decrease in vascular leakage in diabetic mice compared to untreated DR counterparts (Figure 6E-F). Most importantly, retinal dysfunction, a direct consequence of the diabetic state, was found to be significantly mitigated (Figure 6G-I). Collectively, these results suggest that V5 holds considerable promise as a therapeutic intervention for DR, providing benefits that extend beyond vascular normalization, including the restoration of retinal function and integrity of the blood-retinal barrier.

### VED-LYTACs show substantial potential for the treatment of DR

Given the strong inhibitory of V5 on pathological retinal neovascularization and vascular leakage observed in the mouse models of NODs, we next evaluated the potential therapeutic effects of V5 in comparison to existing treatments. We used single injections with equal concentrations and identical treatment durations of V5 and bevacizumab, an available anti-angiogenic agent for angiogenic ocular diseases 50, to compare their effects on retinal neovascularization, vascular leakage, and endothelial cell junction anomalies in DR mice.

**Figure 7:**
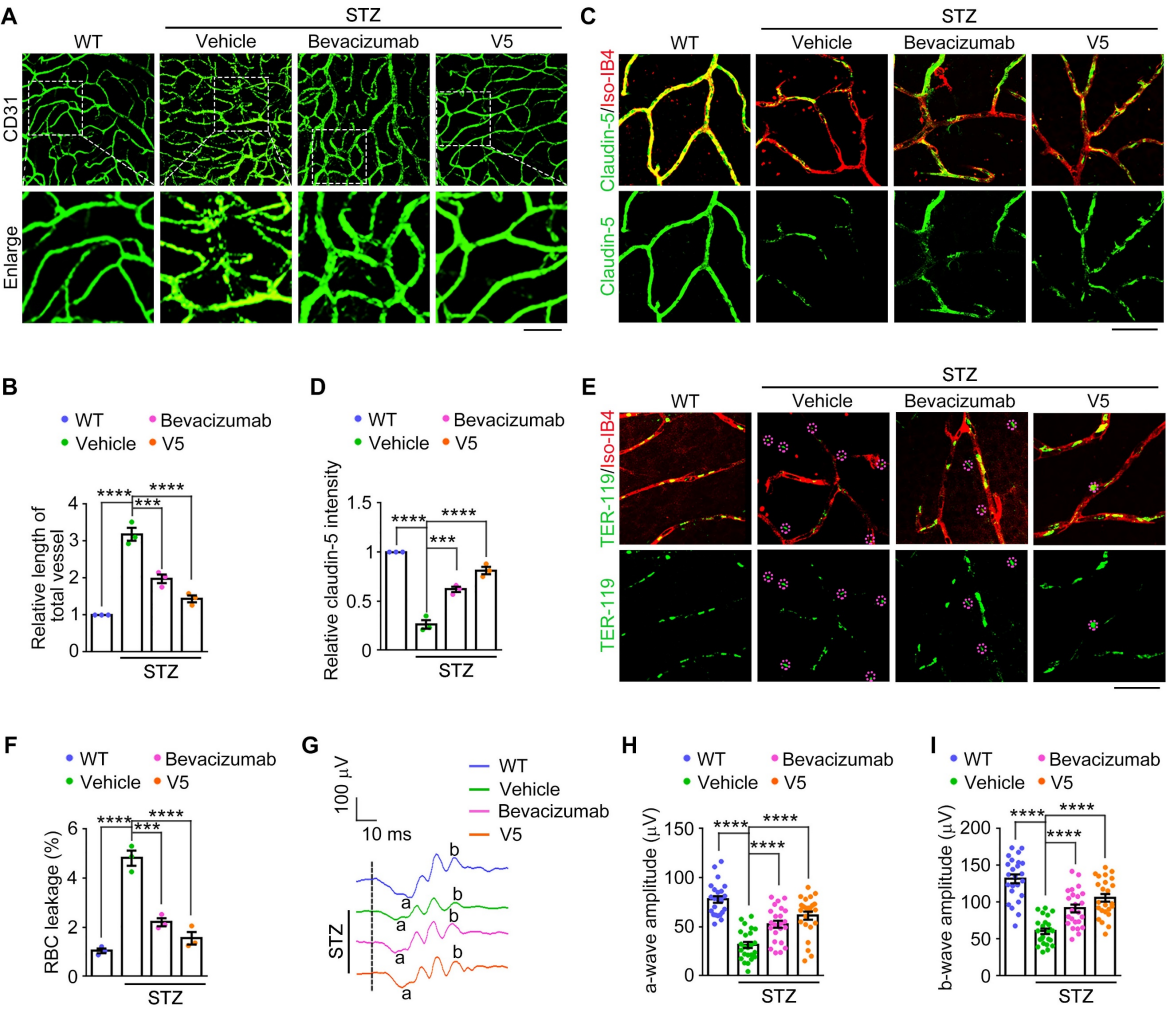
**VED-LYTACs outperform bevacizumab in mitigating vascular pathologies associated with DR. (A and B)** Immunofluorescence images (A) and quantification of the relative retinal vessel length (B, *n* = 3 independent experiments) for the retinal vasculature stained with anti-CD31 in retinas from control or STZ-induced diabetic mice treated with a single injection of 15 µM bevacizumab or V5. Scale bar, 50 µm. **(C and D)** Immunofluorescence images (C) and quantification of the relative claudin-5 intensity (D, *n* = 3 independent experiments) in retinas from control or STZ-induced diabetic mice treated with a single injection of 15 µM bevacizumab or V5, and stained with antibody against claudin-5 and Alexa fluor 568-conjugated Iso-IB4. Scale bar, 20 µm. **(E and F)** Immunofluorescence images (E) and quantification of the percentage of RBC leakage (F, *n* = 3 independent experiments) in retinas from control or STZ-induced diabetic mice injected with a single injection of 15 µM bevacizumab or V5, and stained with antibody against TER-119 and Alexa fluor 568-conjugated Iso-IB4. Purple circles indicate regions of red blood cell leakage. Scale bar, 20 µm. **(G-I)** ERG recordings (G) and measurement of retinal a-wave (H, *n* = 24 eyes from 3 independent experiments) and b-wave (I, *n* = 24 eyes from 3 independent experiments) amplitudes for control or STZ-induced diabetic mice injected with a single injection of 15 µM bevacizumab or V5 under scotopic conditions at 3 cd s m^-2^ flash intensity. Data are presented as mean ± SEM. ***p < 0.001, ****p < 0.0001.

Our comparative analysis revealed that while both V5 and bevacizumab effectively reduced pathological retinal neovascularization and endothelial cell junction anomalies in DR mice, V5 exhibited superior efficacy (Figure 7A-D). Furthermore, both treatments significantly mitigated the vascular leakage typically observed under diabetic conditions, but V5 demonstrated a more pronounced reduction (Figure 7E-F). Additionally, ERG assessments showed that V5 improved retinal function to a greater extent than bevacizumab in DR mice (Figure 7G-I). These findings indicate that V5 is more effective than bevacizumab in ameliorating the vascular pathologies associated with DR. Collectively, these results underscore the substantial potential of V5 as a therapeutic intervention for DR, offering enhanced benefits that include not only superior vascular normalization but also greater restoration of retinal function and integrity of the retinal blood barrier.

## Discussion

Pathological angiogenesis is a primary cause of vision loss in proliferative retinopathies 47. This aberrant vascular growth occurs as a compensatory response to hypoxia and nutrient deprivation, attempting to offset an imbalance in oxygenation and nutrient supply 1. Nonetheless, the consequent neovessels are typically fragile and prone to leakage, potentially leading to retinal detachment. Despite various therapies, approximately 30% of patients still face significant therapeutic challenges 14. Our study introduces a DNA aptamer-based VED-LYTAC system, extensively evaluating its feasibility and probing its therapeutic potential. Employing mouse models of pathological angiogenesis, we substantiated that this innovative VED-LYTAC system can effectively penetrate the murine retinal vasculature and maintain stability. Significantly, our system demonstrated a profound capability in ameliorating pathological vascular proliferation and vascular leakage. These promising results endorse the VED-LYTACs we developed as an emerging candidate with considerable therapeutic prospect for managing angiogenic retinal disorders.

VEGF is well-established as a key regulator of aberrant and excessive blood vessel growth and permeability in the eye, making it a prime therapeutic target for NODs 7,9. This has led to the innovation of numerous therapeutic interventions aimed at VEGF, including those utilizing anti-VEGF strategies based on aptamers 20,51,52. Aptamers are RNA or DNA oligonucleotides engineered through a process known as SELEX (systematic evolution of ligands by exponential enrichment) for their capacity to target proteins with remarkable affinity and specificity 53-55. Due to their distinctive attributes such as minimal toxicity, low immunogenicity, compact size, ease of production, and flexibility, oligonucleotide aptamers have garnered interest for their potential application in disease therapy 56. However, aptamers typically require certain modifications for* in vivo* application due to their naturally small molecular size, which consequently results in relatively low stability within the body. In this study, we selected three DNA aptamers with excellent affinity for VEGF and modified them using a LYTAC-based platform. This transformative approach not only maintained the inherent VEGF-binding proficiency of these aptamers but also conferred upon them the novel function of targeted VEGF degradation both *in vitro* and *in vivo*. Most importantly, this modification significantly enhanced their anti-angiogenic ability, as demonstrated by our data in Figure S3. This advancement represents a significant stride in expanding the therapeutic applications of aptamers, making a promising step toward their clinical integration. These aptamer-based chimeras provide a powerful and precise tool in combating NODs and offer potential for broader application in treating various VEGF-related disorders.

Currently, numerous anti-VEGF therapies are available, but significant challenges remain in the clinical treatment of patients with NODs. In response, we designed novel VED-LYTACs targeting VEGF degradation, diverging from the conventional approach of inhibiting VEGF signaling pathways. This strategy offers distinct advantages. Firstly, the synthesis of VED-LYTACs is more straightforward and cost-effective. Secondly, VED-LYTACs, based on DNA aptamers, exhibit higher biocompatibility and safety. Most importantly, existing anti-VEGF drugs inhibit VEGF by preventing its interaction with VEGFR, but these drugs, typically composed of proteins, peptides, or RNA aptamers, are prone to degradation *in vivo*, necessitating frequent injections. In contrast, VED-LYTACs degrade VEGF during their effective period, addressing this limitation. As demonstrated in Figure 7, a single injection of VED-LYTACs at the same concentration as the widely used bevacizumab shows superior efficacy. These findings highlight the significant therapeutic potential of VED-LYTACs for treating VEGF-related disorders.

In summary, we have demonstrated for the first time that aptamer-based LYTACs can effectively degrade target extracellular proteins in ocular tissues. This chimera forms a ternary complex with M6PR and target VEGF, subsequently transported to lysosomes via the M6PR-mediated pathway, where it undergoes degradation by lysosomal proteases. Our findings suggest that the aptamer-based LYTACs developed in this study offer a potent and promising platform for degrading extracellular proteins, leveraging the advantages of easily synthesized aptamer materials. Future research will focus on modifying aptamers to enhance the *in vivo* stability of these chimeras and exploring new chimera designs to improve the degradation efficiency of targeted proteins.

In this study, we developed an aptamer-based LYTAC system designed to deliver and degrade VEGF specifically in the ocular retina, thereby effectively treating proliferative retinopathy. While our research primarily underscores retinal angiogenesis, the potential of VED-LYTACs to manage pathological angiogenesis in other conditions remains an exciting possibility. Notably, the wet form of age-related macular degeneration (AMD), driven by choroidal neovascularization, is a major cause of vision loss in the elderly in industrialized countries 12. The pathogenesis of wet AMD is attributed to the excessive VEGF production within the retina-choroidal complex, leading to aberrant vascular proliferation and leakage. Given our findings that VED-LYTACs effectively reduce VEGF levels, thereby mitigating VEGF-driven pathological angiogenesis and vascular permeability, we hypothesize that this aptamer-chimera construct could benefit wet AMD. This hypothesis necessitates further validation through the establishment of a laser-induced AMD mouse model in forthcoming studies. Additionally, because VED-LYTACs suppress endothelial cell migration and improve vascular integrity, they might also be beneficial in inhibiting tumor angiogenesis and enhancing vascular stability, which could be particularly relevant as current anti-angiogenic therapies often yield only modest improvements in patient survival due to resistance or therapeutic escape. Assessing whether VED-LYTACs can provide a more prolonged anti-angiogenic effect in cancer therapy could potentially overcome limitations associated with existing treatments.

## Material and Methods

### Animal use and care

All animal experiments were conducted according to the Animal Care and Use Committee of Shandong Normal University (AEECSDNU2021048). Animals had free access to food and water and were exposed to normal lighting conditions with 12-h-on/12-h-off cycles.

### Intravitreal injection

The procedure for intravitreal injection began with anesthesia induction in mice using 2% isoflurane. To alleviate discomfort and facilitate ocular preparation, a drop of 0.5% oxybuprocaine hydrochloride (Santen, Osaka, Japan) was applied to the corneal surface. Pupil dilation was achieved using 0.5% tropicamide with phenylephrine eye drops (Santen), ensuring improved visibility and accessibility for the injection. For intravitreal injection, a 33-gauge Hamilton needle and syringe (Hamilton Company, Reno, NV) was utilized to carefully administer 1 μL of the specified treatment solution into the vitreal space of the eye. Following this delicate procedure, antibiotic treatment was provided as a prophylactic measure against potential infections. Mice with eyeball infections after injection were excluded from the study.

### VEGF-induced retinal angiogenesis

In order to model VEGF-induced retinal angiogenesis, 6-8-week-old mice received an intravitreal injection of 1 μg of VEGF (HZ-1038; Proteintech) using a 33-gauge beveled needle. After 24 h, mice were treated with either 3 μM or 6 μM of the compound V5 for an additional 24 h. Retinas were then harvested for subsequent analysis.

### OIR mouse model

The experimental mouse model of OIR was established in accordance with a rigorously validated protocol as previously 57. In brief, neonatal mice at P7 were placed in a controlled hyperoxia chamber (BioSpherix, Parish, NY), where they were exposed to a consistently maintained atmosphere containing 75% oxygen for a duration of 5 d. Subsequent to this hyperoxic exposure, the mice were returned to normoxic conditions. Starting from P12, intravitreal injections of 1 µL of V5 or vehicle, resulting in final concentrations of 2 µM or 6 µM, were administered every 3 d until the end of the experiment.

### Diabetic mouse model

Male C57BL/6 mice, 8-week-old, underwent a 4 h fasting period before initiation of STZ (dissolved in 0.1 M citrate buffer, pH 4.5; Sigma) administration. Mice received daily intraperitoneal injections of 50 mg/kg STZ or an equivalent volume of citrate buffer as the control for 5 consecutive days. Diabetes induction was confirmed by measuring fasting blood glucose levels using a glucometer 7 d after the final STZ injection. A blood glucose threshold of 16.7 mM or higher was designated as indicative of diabetes. To track the progression and stability of the diabetic state, fasting blood glucose levels were routinely assessed monthly. In parallel, to study the effects of treatment on diabetic mice, intravitreal injections of 1 µL of V5 (the final concentration was 2 µM, 6 µM), or vehicle were administered to the STZ-induced diabetic models over an additional 1-week period with an injection frequency of every 3 d. For the single intravitreal injection, 1 µL of V5 or bevacizumab (HY-P9906, MedChemExpress), at a final concentration of 15 µM, was injected to the STZ-induced diabetic models, and the anti-angiogenesis effects were assessed one week later.

### ERG recording

Prior to initiating the ERG procedure, mice were subjected to a dark adaptation period of 12 h. Following this, the mice were anesthetized, and 0.5% tropicamide ophthalmic solution was applied to induce pupil dilation, along with tetracaine drops for topical analgesia. The handling, preparation, and placement of the electrodes for the ERG recordings were carefully carried out under dim red illumination to preserve the dark adaptation state of the mice. The corneal electrodes were gently positioned after the application of carboxymethyl cellulose with hypromellose, formulated as a comfort-enhancing topical gel to facilitate electrode contact. Scotopic ERGs were systematically captured at light intensity 3 cd sm^-2^. The a-wave amplitude was meticulously quantitated, tracing from the baseline to the bottom of the a-wave, while the b-wave amplitude was calculated from the bottom of the a-wave to the crest of the subsequent b-wave.

### Cell culture and siRNA transfection

Human Umbilical Vein Endothelial Cells (HUVECs) and mouse brain microvascular endothelial cells (bEND.3) were obtained from the American Type Culture Collection (ATCC). These cells were meticulously cultured in specialized endothelial cell medium (ScienCell catalog number 1001) and Dulbecco's Modified Eagle Medium (DMEM, Thermo Fisher), respectively, at an optimal temperature of 37 °C in a humidified chamber with 5% CO_2_. Subconfluent cells, specifically those between passages 2 to 6, were carefully selected for the experiments to ensure optimal growth and response.

For siRNA transfection, the transfection mixture containing siRNAs and Lipofectamine RNAiMAX (Invitrogen) was added into the culture medium without FBS or antibiotics. The medium was changed to fresh endothelial cell medium 12 h after transfection. The sequences of siRNAs used in this study are as follows: Control siRNA (5'-CGUACGCGGAAUACUUCGA-3') and M6PR siRNAs (#1: 5′- UAUAGAGCAAGCCUGGUCU-3′, #2: 5′-AGACCAGGCUUGCUCUAUA-3′). The silencing efficiency was detected by western blotting at 48 h after transfection.

### Synthesis of VED-LYTACs

The preparation of VED-LYTACs began by heating DNA aptamers to 95 °C in 1×DPBS-Mg buffer (DPBS with 12.5 mM Mg (Ac)_2_) followed by rapid cooling on ice. Subsequently, VEGF-A1, VEGF-A2, or VEGF-A3 were combined with M6PR-A in 100 μL of 1×DPBS-Mg buffer and incubated at 4 °C for 30 min to facilitate the complex formation of V1, V2, and V3, respectively. The synthesis of V4, V5, and V6 entailed the addition of T4 ligase to the previous mixtures, with subsequent incubation at 37 °C for 1 h to complete the reaction. To confirm the successful assembly of the VED-LYTACs, the reaction mixtures were subjected to electrophoresis on a 12% native or denatured polyacrylamide gel in 1×TBE-Mg buffer. The gel was run at 120 volts for 90 min, followed by staining with Gel Red to visualize the DNA. The sequences used were provided in Table S1 and were sourced from Sangon Biotech (Shanghai, China) with purification by high-performance liquid chromatography (HPLC).

### Serum stability test

For the serum stability assay, VED-LYTAC compounds were incubated in a medium containing 10% FBS at 37 °C over various time intervals. To assess the integrity of the VED-LYTACs post-incubation, the samples were then electrophoresed using a 12% native polyacrylamide gel prepared with 1×TBE-Mg buffer. This gel electrophoresis was conducted at a constant voltage of 120 volts for a duration of 90 min. Subsequently, the gel was stained using Gel Red staining solution to enable the visualization of the DNA structures, prior to the imaging process.

### Binding affinity analysis

Microscale thermophoresis (MST) assay was performed using a Monolith NT.115 (NanoTemper Technologies GmbH, Munich, Germany). Cy5-labeled VED-LYTACs were prepared to a 5 nM concentration and introduced to varying amounts of VEGF (HZ-1038; Proteintech) or purified GST-M6PR protein from 293T cells in MST buffer containing 20 mM Tris-HCl (pH 8.5), 20 mM KCl, 20 mM CaCl_2_, and 0.1% Tween 20 for 10 min, and then the sample solutions were loaded into capillaries for MST measurements. The normalized fluoresce *F_norm_* ratio for each sample capillary was recorded with time, and 16 samples were measured together. For data analysis, curve fitting and dissociation constant calculations were performed using the MO Affinity Analysis software (NanoTemper Technologies) after three replicate measurements.

### Enzyme-linked immunosorbent assay

The cell culture medium was collected from HUVECs or bEND.3 cells following specific stimulatory treatments. Dissected ocular tissues were finely chopped and lysed in equal volumes of RIPA buffer (Merck) containing 0.1% SDS, protease inhibitor cocktail, and phosphatase inhibitor cocktail (Sigma-Aldrich). The lysates were briefly centrifuged at 1000 rpm for 3 min to remove cellular debris. The resulting clear supernatant was then analyzed using ELISA kits (EK183, EK283, Multi Science), following the manufacturer's detailed instructions, to accurately detect and quantify the presence of VEGF-A.

### Evaluation of lysosomal colocalization

For the colocalization evaluation, HUVECs were cultured on cover slips placed within each well of a 24-well plate. After culture media removal and subsequent dual PBS washes, the prepared cells were treated with labeled aptamers or V5. These compounds were administered in a specialized binding buffer containing 4.5 g/L glucose, 5 mM MgCl_2_, 0.1 mg/mL yeast tRNA, and 1 mg/mL BSA dissolved in PBS at 37 °C, carefully incubated for 30 min. Ensuing treatment, cells underwent a double wash with a washing buffer containing 4.5 g/L glucose and 5 mM MgCl_2_ in PBS. The cells were then exposed to LysoTracker Red (C1046, Beyotime) under optimal conditions of 37 °C, in strict accordance with the supplier's protocol. Post-LysoTracker Red application, two subsequent washes were conducted, followed by cell fixation using 4% formaldehyde for 20 min at ambient temperature. Cell nuclei received staining treatment with DAPI for 5 min. Finalizing the procedure, the cover slides were mounted under dark conditions and visualized using the Dragonfly confocal imaging system (Dragonfly200; Andor Technology, Belfast, UK).

### *In vitro* and* ex vivo* angiogenesis assays

#### Wound-healing assay

The effect of V5 on the migration of HUVECs was evaluated by wound-healing assay. Briefly, HUVECs were cultured in a collagen-coated 6-well plate (2 × 10^5^ cells per well) When cell confluency reached approximately 90%, scratches were created with a sterile 200 μL-pipette in the middle of the HUVECs monolayer. After the cells were washed with PBS 2 times, different concentrations of V5 were added to the wells and cultured in media with 2% FBS for 6, 12, 18 and 24 h. The percentage of wound closure and the relative migration rate between 0 h and 24 h were analyzed with Image J software, based on the wounded area photographed by an inverted microscope.

#### Tube formation assay

Tube formation assay was performed as described previously 58. Briefly, the pretreated HUVECs were seeded in the Matrigel-coated 24-well plate at a density of 2 × 10^5^ cells per well. After 6 h, pictures were taken with a light microscope (Olympus, Tokyo, Japan), and the Angiogenesis Analyser plugin of ImageJ was used to count the total length and the branch points per field.

#### Three-dimensional sprouting assay

For endothelial cell sprouting assays, HUVECs (1×10^3^ cells per well) were cultured with medium containing 0.24% carboxymethyl cellulose in nonadherent round-bottom 96-well plates overnight. Spheroids were collected by centrifugation at 400 g and mixed with 2 mg/mL collagen type I (Corning, USA). Culture medium with or without V5 was then added on top of the gel. Capillary-like sprout formation was examined by microscopy, and angiogenic activity was quantified by measuring the cumulative sprout length per spheroid.

#### Aortic ring sprouting assay

Aortic ring assay was performed following a well-established protocol 42. In brief, thoracic aortas were removed from adult mice, cleaned and treated with V5 for 12 h. The aortas were then embedded in 80 μL matrigel in a 96-well plate containing 500 µL opti-MEM Reduced Serum Media (Gibco) supplemented with 2.5% FBS. Endothelial sprouts were allowed to grow over 5 d with medium containing different concentrations of V5 and changing every other day. Quantification of vessel sprouting was performed by measuring the relative area of aortic explants outgrowth using ImageJ software.

### Immunofluorescence staining

To prepare flat-mounted retinas, mouse eyes were enucleated and fixed in 4% PFA for 1 h at 4 °C. The retinas were then dissected out, washed with phosphate-buffered saline (PBS), and permeabilized in PBS containing 1% Triton X-100 overnight at 4 °C. After permeabilization, the samples were blocked with PBS containing 2% bovine serum albumin (BSA) and 0.3% Triton X-100 for 2 h. To visualize the retinal vasculature, the blocked flat-mounted retinas were incubated with an anti-CD31 primary antibody (MAB1398Z; Millipore) specifically targeting mouse endothelial cells.

For immunofluorescence staining to assess the vascular leakage, retinas were labeled with antibodies against TER-119 (MAB1125; R&D Systems) for red blood cells, claudin-5 (35-2500, Invitrogen) for cell junctions, and Isolectin-IB4 conjugated to Alexa Fluor 568 (I21413, Invitrogen) to stain the vascular network. Subsequent to washing with PBS, the retinas were incubated with Alexa Fluor 488-conjugated secondary antibodies (donkey anti-mouse, A-21202; or donkey anti-rat, A-21208; Thermo Fisher Scientific) for 2 h at room temperature. Following additional PBS washes, nuclei were counterstained with DAPI. The labeled retinas were imaged using either a DM3000 microscope (Leica, Germany) or a Dragonfly200 confocal imaging platform (Andor Technology, Belfast, UK).

To investigate the distribution of Cy5-labeled V5 within the ocular environment, mice underwent an intravitreal injection with either Cy5-conjugated V5 or a non-specific control DNA sequence. Subsequent to various pre-defined time intervals, the mice were anesthetized and prepared for fluorescent imaging utilizing the IVIS spectrum system (PE, Waltham, MA, US). The intensity of the fluorescence was quantitatively measured employing the ROI functionality of the Living Image software. To assess the distribution of V5 in retinal sections, eyes were enucleated from each group and fixed in 4% PFA for 1 h at 4 °C. They were then cryoprotected in 30% sucrose/PBS at 4 °C overnight, embedded in optimal cutting temperature (OCT) compound (Sakura, Japan), and frozen at -80 °C. Serial sections of 10 μm thickness were obtained using a cryostat (CM1950, Leica, Germany). Sections were washed with PBS, permeabilized in 0.3% Triton X-100 in PBS for 15 min, and blocked in 2% BSA with 0.3% Triton X-100 in PBS for 1 h. The sections were incubated with anti-CD31 for 2 h, followed by incubation with Alexa Fluor 488. Images were captured on a Dragonfly200 confocal imaging platform (Andor Technology, Belfast, UK). The fluorescence intensity of Cy5-labeled V5 in CD31 positive areas was quantified using ImageJ software.

### Histopathological analysis

Eye samples were fixed in 4% paraformaldehyde overnight at 4 °C. Following fixation, the cornea and lens were removed, and the retinas were further fixed for an additional 2 h at room temperature. The retinas were then embedded in paraffin, and 4 mm sections were prepared. Sections including the optic nerve were chosen for hematoxylin and eosin (H&E) staining using a previously described standard protocol 59. For measuring retinal thickness, sections from the same eccentricity and eye cups embedded in the same orientation were selected. Images were captured and analyzed using a DM3000 microscope (Leica, Wezlar, Germany).

### Immunoblotting and streptavidin pulldown

Proteins were resolved with cold lysis buffer containing Tris (50 mM, pH 7.5), NaCl (150 mM), EDTA (1 mM), glycerin (3%), NP40 (1%), and complete protease inhibitor tablets (Roche, Basel, Switzerland). Lysates were cleared by centrifugation at 15 000 rpm for 20 min at 4 °C. Supernatants were added with SDS sample buffer (pH 6.8) containing urea (8 M) and subjected to 8% SDS-PAGE. Proteins were transferred to polyvinylidene difluoride membranes (Millipore) and subjected to immunoblot analysis. For streptavidin pulldown, cell lysates were incubated with the indicated aptamers dissolved in DPBS-Mg buffer at a 1:1 ratio, VED-LYTACs or biotin at 4 °C for 4 h, followed by incubating with streptavidin agarose beads (20353; Thermo Fisher Scientific) for another 2 h. The beads were pelleted and immunoblotted with the indicated antibodies. Primary antibodies for immunoblotting were as follows: GFP (11814460001; Roche), VEGF (19003-1-AP; Proteintech), M6PR (ab124767; Abcam), and *β*-actin (A5316, Sigma-Aldrich).

### Statistical analysis

Statistical evaluations were performed using the GraphPad Prism 8.0 software suite (developed by GraphPad Software, La Jolla, CA). All experimental findings were presented as the mean ± SEM. In cases where the data conformed to a normal distribution, student's* t*-test was employed to discern differences between two distinct groups, whereas a two-way analysis of variance (ANOVA) was utilized for comparisons involving three or more groups. On the other hand, for data sets that deviated from normal distribution, nonparametric statistical methods were applied. In this analytical context, significance was ascribed to p-values that < 0.05.

## Supplementary Material

Supplementary figures and table.
