# Supplementary material for: Targeted degradation of VEGF with bispecific aptamer-based LYTACs ameliorates pathological retinal angiogenesis
Source: Theranostics. 2024 Aug 19;14(13):4983–5000. doi: 10.7150/thno.98467 (PMC11388081; doi:10.7150/thno.98467)
Supplement: Supplementary file 1 — Supplementary figures and table. [file thnov14p4983s1.pdf]

Supplementary information

Targeted degradation of VEGF with bispecific aptamer-based LYTACs ameliorates pathological retinal angiogenesis

Ping Zhou[#], Sai Zhang[#], Lin Li[#], Renshuai Zhang, Guizhi Guo, Yufei Zhang, Runa Wang, Miaoyuan Liu, Zhiyi Wang, Huijie Zhao, Guiwen Yang, Songbo Xie, Jie Ran*

Center for Cell Structure and Function, Shandong Provincial Key Laboratory of Animal Resistance Biology, College of Life Sciences, Shandong Normal University, Jinan 250014, China.

[#]These authors contributed equally to this work.
*Correspondence: jran@sdnu.edu.cn (J.R.)

Table S1

Name	Sequence (5'-3')
M6PR aptamer	GGCGCGTAGATGACGAGCAGTCCTAACATCGTTTAGGAC
VEGF aptamer-1	TGTGGGGGTGGACTGGGTGGGTACC
VEGF aptamer-2	TGTGGGGGTGGACGGGCCGGGTAGA
VEGF aptamer-3	GCCCGTCTTCCAGACAAGAGTGCAGGGC
M6PR-A for V1, V2 and V3	CGTAAATCAGTCATAGGGCGCGTAGATGACGAGCAGTCCTAACATCGTTTAGGAC
M6PR-A for V4, V5 and V6	*P*-CCCCCACACGTAAATCAGTCATAGGGCGCGTAGATGACGAGCAGTCCTAACATCGTTTAGGAC
VEGF-A1 for V1	TATGACTGATTTACGTGTGGGGGTGGACTGGGTGGGTACCC
VEGF-A2 for V2	TATGACTGATTTACGTGTGGGGGTGGACGGGCCGGGTAGA
VEGF-A3 for V3	TATGACTGATTTACGGCCCGTCTTCCAGACAAGAGTGCAGGGC
VEGF-A1 for V4	TATGACTGATTTACGTGTGGGGGTGGACTGGGTGGGTACCC
VEGF-A2 for V5	TATGACTGATTTACGTGTGGGGGTGTGGGGGTGGACGGGCCGGGTAGA
VEGF-A3 for V6	TATGACTGATTTACGTGTGGGGGGCCCGTCTTCCAGACAAGAGTGCAGGGC

The sequences of aptamers (M6PR aptamer and VEGF aptamer) are underlined. The linkers (15-bp and 23-bp) of the corresponding VED-LYTACs (V1-V6) are labeled with red color.

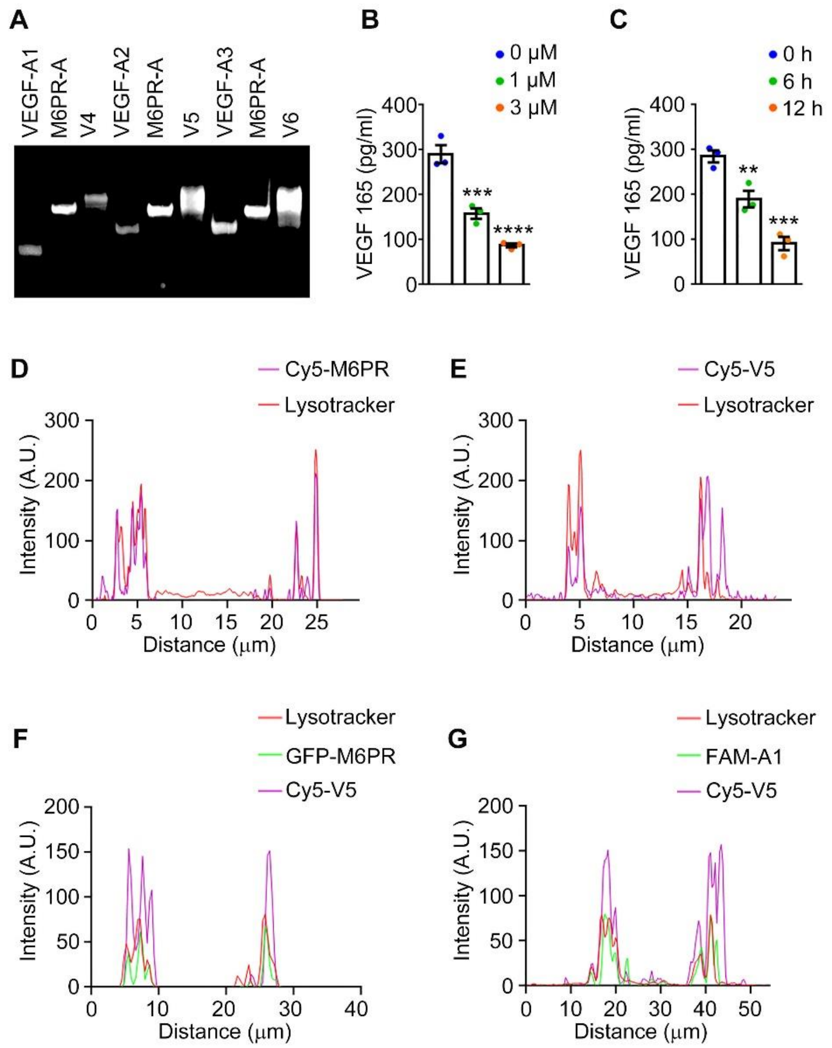

Figure S1. Construction of VED-LYTACs and evaluation of its ability to degrade VEGF. (A) Denatured polyacrylamide gel analysis of the V4, V5 and V6. **(B)** ELISA assay for analysis the VEGF level from bEND.3 cells treated with different concentrations of V5 for 12 h (n = 3 independent experiments). **(C)** ELISA assay for analysis the VEGF level from bEND.3 cells treated with V5 for 6 h or 12 h (n = 3 independent experiments). **(D and E)** Quantifications of fluorescence intensity for lysosome colocalization analysis in HUVECs treated with Cy5-labeled individual aptamers or V5. **(F and G)** Quantification of fluorescence intensity for lysosome colocalization analysis in HUVECs treated with Cy5-labeled V5, along with GFP-M6PR (F) or FAM-A1 aptamer (G) to label M6PR or VEGF proteins.

Data are presented as mean ± SEM. **$p < 0.01$, ***$p < 0.001$, ****$p < 0.0001$.

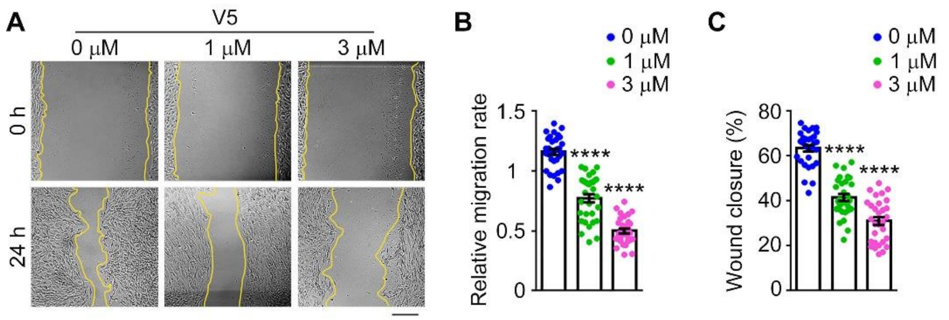

Figure S2. VED-LYTACs inhibit cell migration in bEND.3 cells. (A-C) Representative images (A) and quantifications of the relative migration rate (B, n = 30 areas from 3 independent experiments) and percentage of wound closure (C, n = 30 areas from 3 independent experiments) for the scratch migration assay in bEND.3 cells treated with different concentrations of V5. Scale bar, 80 μm.

Data are presented as mean ± SEM. ****p < 0.0001.

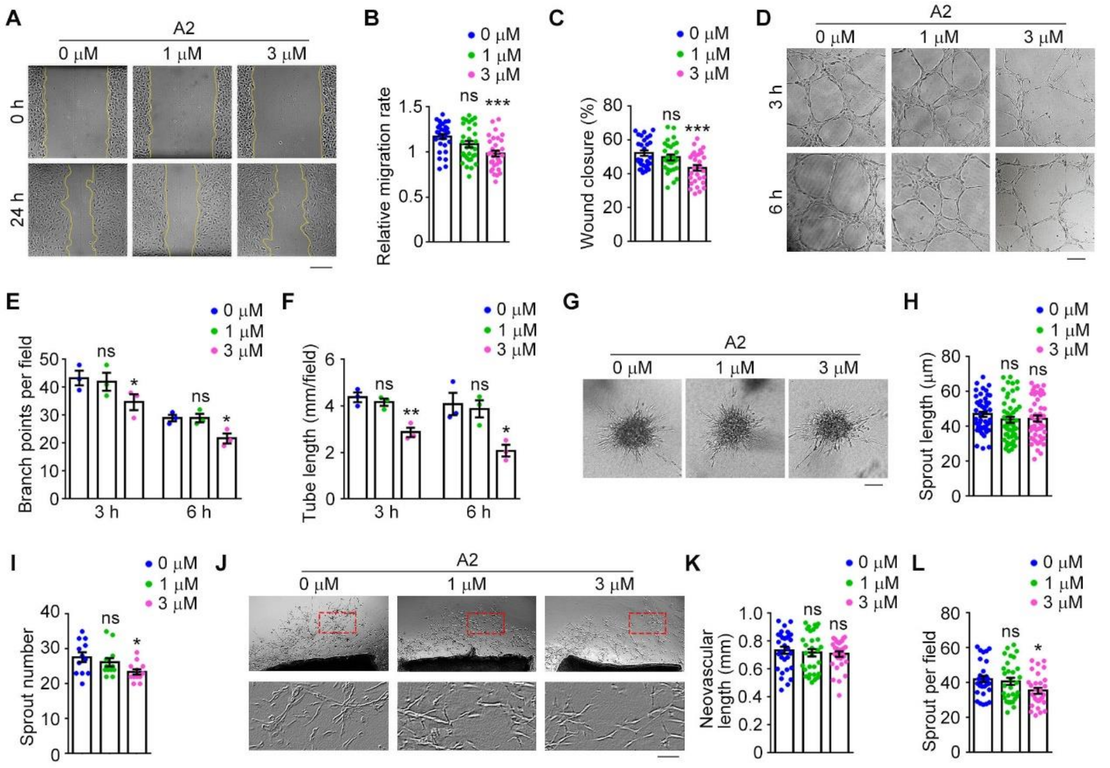

Figure S3. A2 aptamer exhibits less efficacy in inhibiting angiogenesis compared to V5. (A-C) Representative images (A) and quantifications of the relative migration rate (B, n = 30 areas from 3 independent experiments) and percentage of wound closure (C, n = 30 areas from 3 independent experiments) for the scratch migration assay in HUVECs treated with different concentrations of A2. Scale bar, 80 μm. **(D-F)** Representative images (D) and quantifications of the branch points (E, n = 3 independent experiments) and the tube length (F, n = 3 independent experiments) for tube formation assay in HUVECs treated with different concentrations of A2. Scale bar, 200 μm. **(G-I)** Representative images (G) and quantifications of the total sprout length (H, n = 50-80 sprouts from 3 independent experiments) and numbers (I, n =12 spheroids from 3 independent experiments) for sprouting assay from HUVECs treated with

different concentrations of A2. Scale bar, 30 μm. **(J-L)** Representative images (J) and quantifications of the neovascular length (K, n =30 fields from 3 independent experiments) and the sprout numbers per field (L, n =30 fields from 3 independent experiments) for sprouting assay from mice aortic ring treated with different concentrations of A2. Scale bar, 300 μm.

Data are presented as mean ± SEM. *$p < 0.05$, **$p < 0.01$, ***$p < 0.001$; ns, not significant.

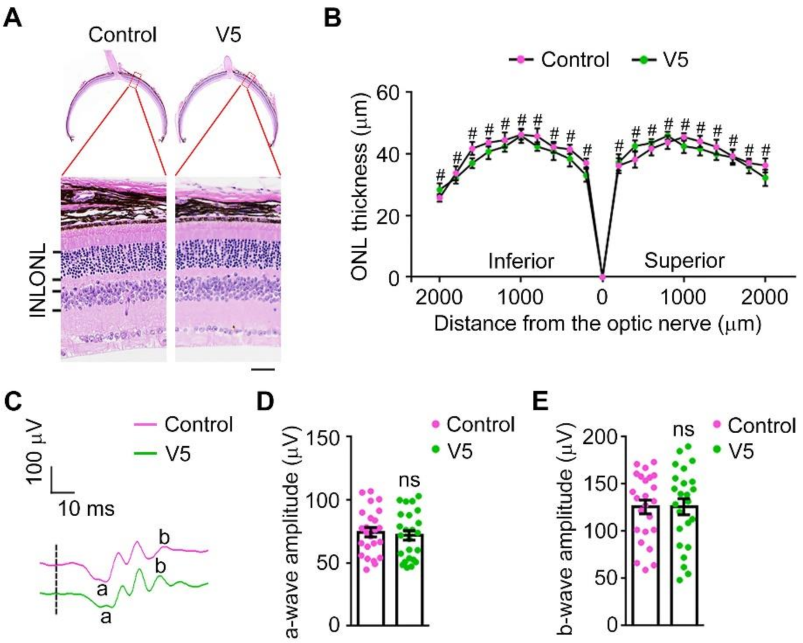

Figure S4. The biosafety of VED-LYTACs in an ocular application. (A and B) Photomicrographs (A) and quantification (B) of the retinal histology assessed by hematoxylin and eosin (H&E) staining in control and V5-injected mice (6 μM, n = 12 mice from three independent experiments). ONL, outer nuclear layer. **(C-E)** ERG recordings (C) and measurement of retinal a-wave (D, n = 24 eyes from 3 independent experiments) and b-wave (E, n = 24 eyes from 3 independent experiments) amplitudes for control and V5 (6 μM)-injected mice under scotopic conditions at 3 cd s m^{-2} flash intensity.

Data are presented as mean ± SEM. # and ns, not significant.

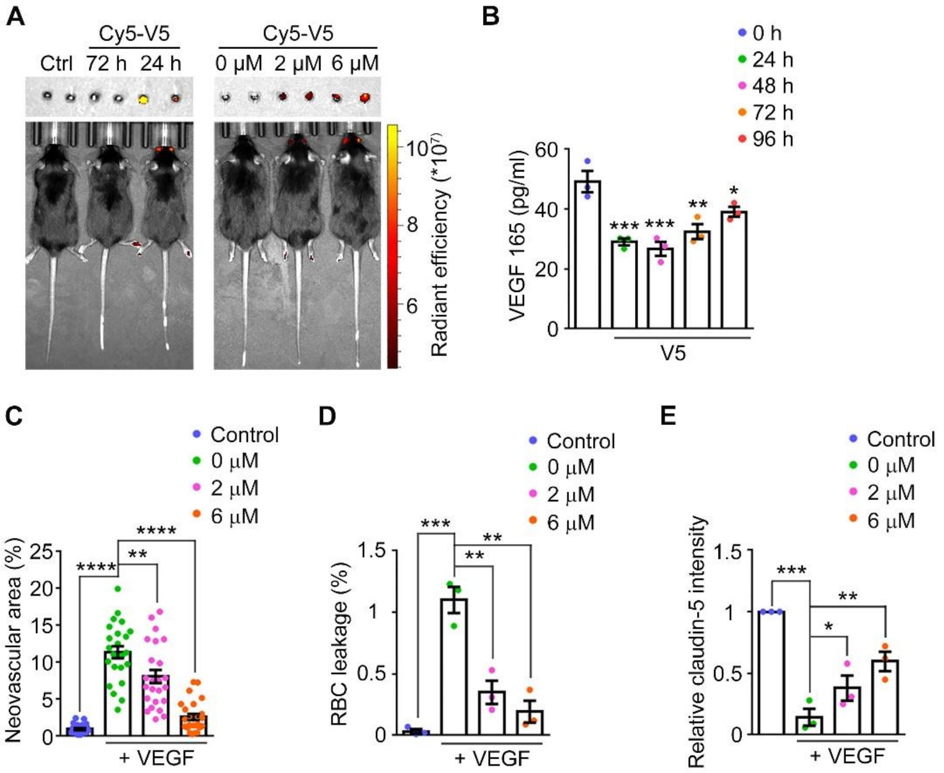

Figure S5. Anti-angiogenic effect of V5 *in vivo*. (A) Images of mice injected with 2 μM of Cy5-V5 for the indicated times (left) or different concentrations of Cy5-V5 for 48 h (right) using the *in vivo* imaging system. **(B)** ELISA assay for analysis the VEGF level from retinas treated with 6 μM of V5 for indicated times (n = 3 independent experiments). **(C)** Quantification of the percentage of the neovascular area in retinas from control or VEGF-injected mice treated with different concentrations of V5 (n = 30 fields from 3 independent experiments). **(D)** Quantification of the percentage of RBC leakage in retinas from control or VEGF-injected mice treated with different concentrations of V5 (n = 3 independent experiments). **(E)** Quantification of the relative claudin-5 intensity in retinas from control or VEGF-injected mice treated with different concentrations of V5 (n = 3 independent experiments).

Data are presented as mean ± SEM. $*p < 0.05$, $**p < 0.01$, $***p < 0.001$, $****p < 0.0001$.

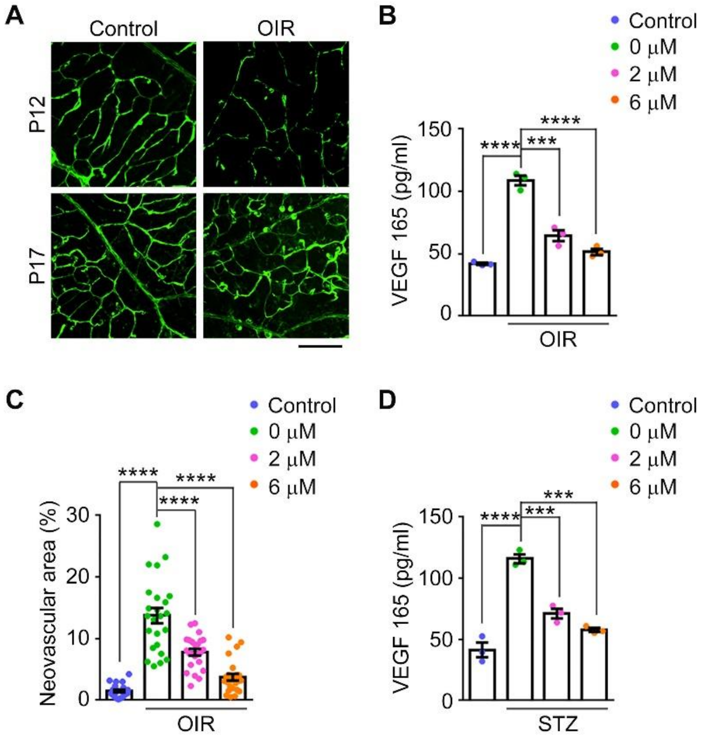

Figure S6. Anti-angiogenic effect of V5 in OIR mouse models. (A) Immunofluorescence images of the retinal vasculature stained with anti-CD31 in retinas from control or OIR mice. Scale bar, 30 μm. **(B)** ELISA assay for analysis the VEGF level in retinas from control or OIR mice treated with different concentrations of V5 (*n* = 3 independent experiments). **(C)** Quantification of the percentage of the neovascular area in retinas from control or OIR mice treated with different concentration of V5 (*n* = 30 fields from 3 independent experiments). **(D)** ELISA assay for analysis the VEGF level in retinas from control or STZ-induced diabetic mice treated with different concentrations of V5 (*n* = 3 independent experiments).
Data are presented as mean ± SEM. $***p < 0.001$, $****p < 0.0001$.
